# Minimum spanning tree analysis of EEG resting-state functional networks in schizophrenia

**DOI:** 10.1038/s41598-024-61316-8

**Published:** 2024-05-07

**Authors:** Melinda Becske, Csilla Marosi, Hajnalka Molnár, Zsuzsanna Fodor, Kinga Farkas, Frigyes Sámuel Rácz, Máté Baradits, Gábor Csukly

**Affiliations:** 1 Department of Psychiatry and Psychotherapy, Semmelweis University, Balassa u. 6., Budapest, 1083 Hungary; 2 Department of Physiology, Semmelweis University, Budapest, Hungary

**Keywords:** Cognitive neuroscience, Schizophrenia

## Abstract

Schizophrenia is a serious and complex mental disease, known to be associated with various subtle structural and functional deviations in the brain. Recently, increased attention is given to the analysis of brain-wide, global mechanisms, strongly altering the communication of long-distance brain areas in schizophrenia. Data of 32 patients with schizophrenia and 28 matched healthy control subjects were analyzed. Two minutes long 64-channel EEG recordings were registered during resting, eyes closed condition. Average connectivity strength was estimated with Weighted Phase Lag Index (wPLI) in lower frequencies: delta and theta, and Amplitude Envelope Correlation with leakage correction (AEC-c) in higher frequencies: alpha, beta, lower gamma and higher gamma. To analyze functional network topology Minimum Spanning Tree (MST) algorithms were applied. Results show that patients have weaker functional connectivity in delta and alpha frequency bands. Concerning network differences, the result of lower diameter, higher leaf number, and also higher maximum degree and maximum betweenness centrality in patients suggest a star-like, and more random network topology in patients with schizophrenia. Our findings are in accordance with some previous findings based on resting-state EEG (and fMRI) data, suggesting that MST network structure in schizophrenia is biased towards a less optimal, more centralized organization.

## Introduction

Cortical dysconnectivity is often regarded as a core dysfunction in schizophrenia^1^. Altered connectivity between different brain areas is related to aberrant synaptic plasticity caused by anomalies in multiple neurotransmitter systems^2–4^. Functional connectivity refers to statistical associations between neurophysiological time series data of remote neural populations^5–7^. By analyzing resting-state EEG it is possible to investigate intrinsic, spontaneous functional connectivity in a task-free condition^8^.

Regarding the strength of functional connectivity between remote brain areas, quite heterogeneous results have been found in schizophrenia: both increased and decreased as well as unchanged connectivity have been observed during rest in patients, depending on the areas analyzed^2,9^. Various methods (phase-based^8,10–13^ and amplitude-based^14^) have been used to compute coherence^15,16^ or correlation^14,17^ between data registered from different brain areas either on the source level^8,14,16^ or on an electrode level^10,11,13,17^.

Concerning the delta frequency band, previous studies generally found no difference in the strength of connectivity in patients compared to controls at the electrode level^11,18^, or alternatively, weaker delta connectivity was found in the patient group^19^. On the other hand, either no difference^18^ was found in the theta band or increased^11^, or even decreased connectivity^17^ was reported in patients with schizophrenia. Besides these, lower connectivity strength was reported in patients in the alpha band in a number of articles and it appears to be the most stable and robust result across studies^2,9,11,17,18^. While typically no difference was found in the beta band^2,11^, results are somewhat ambiguous, as besides no difference, both increase and decrease in beta band connectivity have been found^2,13,18^. In gamma, the results are not clear, they seem to depend on the method used to quantify the strength of connectivity and on the examined areas^2,8,9,13,14,17^.

In general, some findings suggest the presence of decreased functional connectivity in delta and alpha bands, and increased connectivity in the beta and gamma bands in patients with schizophrenia^19^, however the work of Olejarczyk & Jernajczyk^19^ also shows that the results obtained can be dependent on the choice of connectivity measure and reference electrode, and the heterogeneity of results can as well be partly caused by heterogeneity (in terms of demographic and clinical factors) of the patient groups enrolled. Evidence of weaker functional connectivity strength was also found in a number of studies using functional MRI^20–22^.

In order to explore the overall patterns of whole-brain functional connectivity—i.e. communication patterns between remote brain areas—graph-theoretical analyses are applied. Recently, the Minimum Spanning Tree (MST) approach^23,24^ is becoming increasingly widely used as it allows a simplified, and an unbiased network representation, making it more appropriate for the comparison of networks obtained from distinct populations of subjects, networks differing in density^20,25^. However, since MST graphs do not contain any loops, certain aspects of network organization, described in this paper (such as clustering and modularity), cannot be directly examined^24^.

Regarding global network organization, two extremes can be distinguished: path-like (or line-like) and star-like topology^26^. In a path-like (segregated, i.e. minimally integrated) network, all nodes are linked to two other nodes, except for the two end nodes that only have one connection. These nodes are referred to as the leaves. In a star-like (maximally integrated) configuration, on the other hand, all nodes except for one are linked to a central node^26^. In this example, we have many leaf nodes and one central hub node. Between these two extremities, different configurations can occur such as healthy brain topology.

The network organization in a healthy brain is characterized by small-worldness and modularity. Small-worldness refers to the balance between local segregation (selective, region-specific information processing) and global integration (convergent information processing) that ensures the most efficient information flow between brain areas with minimum cost^26,27^. Modules are functionally specialized groups of nodes that are densely intraconnected and sparsely interconnected with nodes of other modules in the network. These modules are hierarchically organized, and the efficient communication between specialized and relatively segregated modules is ensured by some prominent hubs that are likely to form connections with each other (“rich clubs”)^27^. In the optimal network, segregation and integration processes are balanced out, a hierarchical structure emerges, where the presence of relatively low number of leaf nodes prevents from hub overloading, and at the same time, multiple central or prominent hub nodes create rich clubs for efficient information flow^26^.

This modular, hierarchical, balanced, cost-efficient organization of the functional network (e.i. small-world topology with rich clubs) ensures optimal information processing in the healthy brain. However, different neurological and psychiatric conditions are characterized by distinct patterns of altered connectivity and biased network topology^27^. The balance between segregation and integration have been found to be compromised in diseases as ADHD^28^, Multiple sclerosis^29^, Major Depressive Disorder^30^, Bipolar disorder^20^, Alzheimer’s disease^31^, and schizophrenia^11^. Network analysis has been deemed particularly useful for diagnostic purposes in dementia and epilepsy^27^.

Previous results, however, regarding functional network topology in patients with schizophrenia are mixed. Both disrupted integration (decentralization)^32,33^, and increased integration (centralization)^10–12,19,22,34^ have been found in patients with schizophrenia. This heterogeneity may be partly accounted for by methodological issues and various differences between the studies and patient groups^19^. However, the most recent results using the unbiased MST method tend to point to higher global integration, centralization in schizophrenia (i.e. a more star-like topology with many leaf nodes and a few overloaded hubs)^11^. Increased randomness is also often found in the patient population, which means that the formation of rich clubs is less likely as central nodes tend to be linked to leaf nodes directly. It is associated with dysmodularity, disturbance of the modular organization in the network topology of patients^17,22,34,35^.

In line with these findings, we hypothesized that the global functional network configuration of patients with schizophrenia would be biased towards integration. Based on the literature, we further hypothesized that the overall average connectivity strength would be weaker in the patient group, especially in the alpha frequency band. Schizophrenia is a characterized by serious executive deficit^36^ that has been found to be related to defrontalization^36^, inspired by the work of^11^, we have also decided to compare average values of betweenness centrality (i.e. an indication of global importance) of anterior and posterior nodes—along with global average values of node importance—between the two groups. In order to get further insights regarding the nature of functional network abnormalities in schizophrenia, we have also analysed the randomness of the network in terms of increased disassortativity.

## Materials and methods

### Participants

The study took place in the Department of Psychiatry and Psychotherapy, Semmelweis University, Budapest, Hungary. EEG was recorded from 37 patients with schizophrenia and 37 healthy control participants during resting but due to artifacted recordings, data of 32 patients (male = 31.3%, average age = 33.2, SD = 10.8) and 28 controls (male = 40.7%, average age = 34, SD = 10.2) were included in the analysis. Demographic and clinical data are shown in Table 1**.**

**Table 1:** Demographic data of the study groups, and clinical information of the patient group.

	Patients	Controls	Statistics	*p*-value
	Group (n = 32)	Group (n = 28)		
	Mean (SD)	Mean (SD)		
Gender (male %)	31.3%	40.7%	Chi2 = 0.58	0.448
Age (years)	33.22 (10.78)	34.04 (10.24)	t = 0.299	0.766
Education level (%)*	9.4%/59.4%/31.2%	0%/66.7%/33.3%	Fisher’s exact test	0.396
Illness duration (years)	7.59 (8.33)	–		
CPZ equivalent dose (mg)	447.8 (353.3)	–		
PANSS total	64.13 (19.51)	–		
PANSS positive	15.07 (4.96)	–		
PANSS negative	16.7 (6.06)	–		
PANSS general	32.37 (10)	–		

*Education level: 1 = elementary school/ 2 = high school/ 3 = college/university. CPZ = chlorpromazine equivalent dose. PANSS = Positive and Negative Symptoms Scale.

The study was approved by the Regional and Institutional Committee of Science and Research Ethics, Semmelweis University, Budapest, Hungary (registration number: 197/2015, date: October/05/2015). Participants gave their written informed consent before the procedures. The experiments were carried out in full compliance with the Helsinki Declaration.

### EEG recording and processing

During EEG examinations participants were seated in a dimly lit, sound-attenuated room. EEG was recorded from DC using a 64-channel Neuroscan amplifier. Due to huge artifacts, 9 channels were eliminated. The analyzed channels were: FP1, FPZ, FP2, AF3, AF4, F7, F5, F3, Fz, F4, F6, F8, FT7, FC5, FC3, FC1, FC2, FC4, FC6, FT8, T7, C5, C3, C1, CZ, C2, C4, C6, T8, CP5, CP3, CP1, CPZ, CP2, CP4, CP6, P7, P5, P3, P1, Pz, P2, P4, P6, P8, PO7, PO5, PO3, POZ, PO4, PO6, PO8, O1, Oz, O2. Electrode caps had an equidistant layout and covered the whole head according to the Neuroscan montage. Eye movements were monitored with EOG electrodes placed below the left and above the right external canthi. Data were digitized at a sampling rate of 1000 Hz. Built-in and self-developed functions as well as the freeware EEGLAB toolbox^37^ in the Matlab (MathWorks, Natick, MA) development environment was used for subsequent off-line data analyses. The 2-min EEG segments were evaluated for huge artifacts. As all subject’s data segments had to be of the same length, and the channels chosen for the analysis also had to be the same (since interpolation was not used), we set some limits prior to data inspection: participants, who did not have at least seven usable 8 s long segments (segments with no large artifacts) were to be rejected. Recordings were inspected by two investigators independently. Rejection was done based on visual inspection. Segments without huge artifacts were marked. Data was then filtered between 0.3 and 200 Hz, using zero-phase shift forward, and reverse IIR Butterworth filter. Narrow band stop filters were also applied for the following frequency ranges: 49.5–50.5 Hz, 99.5–100.5 Hz, 149.5–150.5 Hz, 199.5–200.5 Hz. After that, EEG was epoched to 8 s segments, and ICA (Independent Component Analysis) was applied to the epoched recordings. This way, the same components were removed across segments. Data was resampled to 512 Hz. Automatic artifact removal was done with MARA (Multiple Artifact Rejection Algorithm)^38^ to remove muscle, blinking, and eye movement artifacts. After artifact rejection, EEG was re-referenced to the common average, and uniformly 7–7 clean 8 s long epochs were selected for each participant. After applying MARA, on average, approximately 20 independent components remained in the EEG data, and no significant between-group difference was found in the number of independent components after artifact rejection (t = − 0.99; *p* = 0.324). As a final step, clean data were exported in asci format for analyses.

### EEG data analysis

After artifact rejection, EEG connectivity analyses were performed with open-access software BrainWave (version 0.9.152.12.26; available at http://home.kpn.nl/stam7883/brainwave.html [accessed on 18 May 2021]) on epochs of 8-s duration (sampling rate 512 Hz, 4096 time points). In low-frequency bands (delta and theta), the strength of functional connectivity between each EEG channel was analyzed by weighted phase-lag index^39^. For higher frequency bands (alpha, beta, low and high gamma) connectivity strength was evaluated by measuring the amplitude envelope correlation with leakage correction (AEC-c)^6^ calculated for all EEG epochs of each subject, after having band-pass filtered the EEG time-series in the delta (0.5–4 Hz), theta (4–7 Hz), alpha (7–13 Hz), beta (13–30), lower gamma (30–48 Hz) and higher gamma (52–70 Hz) frequency bands.

The Phase Lag Index measures phase synchronization based on the asymmetry of the distribution of instantaneous phase differences between two signals^40^. This distribution is weighted by the magnitude of the imaginary component of the cross-spectrum in the weighted version of the PLI. Previous research found that the weighted version of the PLI is superior to the original metric in finding connections between EEG time series data, as it is less sensitive to noise and better controls for the effect of volume conduction (for further details, please see the study on the weighted Phase Lag Index by Vinck and colleagues^39^). The leakage-corrected version of the Amplitude Envelope Correlation (AEC-c) measures the linear correlation of the envelopes of band-pass filtered signals by first applying pair-wise symmetric orthogonalization (linear regression analysis) to the time-series data, in order to remove zero-lag correlations caused by volume conduction^6^.

Connectivity metrics were averaged over epochs for each participant. Global functional connectivity values were calculated by averaging connectivity strength of all electrodes. The choice of using a phase-based connectivity measure in lower frequencies, and a correlation-based metric in medium and higher frequency bands was motivated by a recent work of Briels and colleagues^5,41^. It was found by them that AEC-c outperformed PLI in terms of validity and reproducibility in higher frequency bands (alpha and beta) but PLI showed reproducible effects in the theta band in Alzheimer’s disease.

To determine epoch length, we relied on the literature. Previous data shows that MST parameters stabilize at 1–6 s if the MST is based on PLI, and at 4–8 s if it is based on AEC^42^. Furthermore, 8-s segments were used in a similar study by Krukow and colleagues^11^ as well.

### Graph-theoretical analysis

In order to analyze global functional network characteristics in the two study groups, the graph-theoretical representation of the functional connectivity matrix was created by the Minimum Spanning Tree (MST) algorithm. MST is a simplified representation of the core network containing the strongest and most relevant connections, where all nodes (in our case, electrodes) are connected without forming loops^23,26^. The advantage of the MST approach lies in the fact that it overcomes the bias of network density and degree making it more suitable for between-group (e.g. patient vs. control) comparisons^11,20^. MST graphs were generated for each participant, each analyzed frequency band, and epoch separately, based on the connectivity matrices (wPLI, AEC-c) previously obtained for each pair of electrodes.

**Figure 1:**
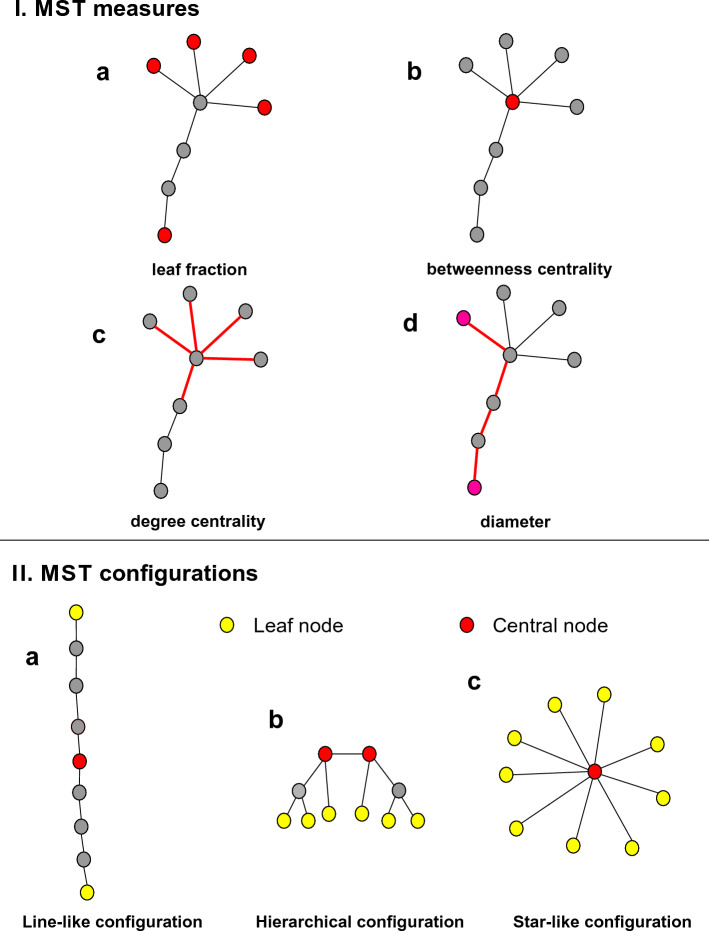
Schematic representation of MST measures (I.) and MST structures (II.). **(I. a)** *Leaf fraction:* the number of nodes with only one connection divided by the total number of nodes of the tree. **(I. b)** *Betweenness centrality:* maximum betweenness centrality is the fraction of all shortest paths that pass through the most important node for global communication in the tree. **(I. c)** *Degree centrality:* maximum degree is the highest number of edges connected to a node in the tree. **(I. d)** *Diameter:* the longest distance (i.e. maximum number of edges) between any two nodes of the network normalized by the total number of connections in the tree^44^. **(II. a)** The extreme ***line-like*** (minimally integrated) network configuration is characterized by *low leaf fraction*, *low betweenness centrality*, and *high diameter*. Segregation processes are dominant in such networks. This type of network is inefficient as it takes many steps to transfer information from one node to another. **(II. c)** The extreme ***star-like*** configuration (maximally integrated), on the other hand, is characterized by *high leaf fraction*, *high betweenness centrality*, and *low diameter*. Although this network is efficient, the central hub can become overloaded, and can fail as a result. **(II. b)** At the center, the intermediate, balanced ***hierarchical***, modular configuration is regarded as the optimum, as it ensures effectivity (*relatively low diameter*) while, simultaneously protects against hub overload (*relatively low betweenness centrality* and *leaf fraction*). Also, in case of a more resilient, *less diassortative* network, highly connected nodes are likely to be connected to each other, creating *“rich clubs”*^11,26,34^*.*

Although, a number of parameters can be computed from the MST graph, these parameters are somewhat redundant as most of them are highly correlated to each other. For this reason, we have chosen to concentrate on the analysis of four measures: *diameter*, *leaf fraction*, *maximum degree centrality* and *maximum betweenness centrality* (measures of functional integration)*, and assortativity* (as measure of randomness and network resilience). The *diameter* is the longest distance (i.e. maximum number of edges) between any two nodes of the network normalized by the total number of connections in the tree. Low diameter means that information spreads efficiently across remote nodes. *Leaf fraction* is the number of nodes with only one connection divided by the total number of nodes of the tree. Diameter is inversely related to leaf number, so it decreases when leaf number increases. *Degree* is computed for each node, and it refers to the number of edges connected to the node. The nodes with high degree are referred to as hubs. Degree of the node with the highest degree (*maximum degree centrality*) gives the strength of the most important node in the network. *Betweenness centrality* (BC) was also computed for each node. It is the fraction of all shortest paths that pass through a particular node. *Maximum BC* indicates the importance of the most central node (the node most important for global communication). It is a measure of centrality of the network organization^23,26^. Low diameter, high leaf fraction, and high maximum betweenness centrality suggest elevated integration processes, and a more centralized, star-like network organization^26^ (Fig. 1).

We also decided to analyze network *assortativity*, to compare the amount of randomness of the network structure between the two study groups. Assortativity refers to the correlation between node degrees. The more negative the correlation, the more dissassortative (randomly organized) the network structure is^43^.

Besides maximum BC, *global average BC* (mean BC of all channels) was also computed, and *average regional BC* was assessed separately for *anteriorly* (FP1, FPZ, FP2, AF3, AF4, F7, F5, F3, FZ, F4, F6, F8, FT7, FC5, FC3, FC1, FC2, FC4, FC6, FT8) and *posteriorly* (P7, P5, P3, P1, PZ, P2, P4, P6, P8, PO7, PO5, PO3, POZ, PO4, PO6, PO8, O1, OZ, O2) located channels as well ^11^.

MST parameters were computed with the Brainwave software (version 0.9.152.12.26; available at http://home.kpn.nl/stam7883/brainwave.html). Network parameters were averaged across epochs.

### Statistical analysis

EEG variables were compared between the study groups with Welch independent samples t-tests. Statistical significance was determined at *p* < 0.05. FDR correction for multiple comparisons was applied^45^ simultaneously to all frequency bands (delta, theta, alpha, beta, low and high gamma) and connectivity/MST measures (e.i. global measures: average connectivity, leaf fraction, degree centrality, betweenness centrality, diameter, and assortativity), except for the exploratory analyses (regional analyses). To carry out the correction, we used the R implementation of the algorithm (package: „stats”, function: „p.adjust”). To characterize the magnitude of the effects we reported the values of effect size in terms of Cohen’s d.

## Results

### Functional connectivity

**Figure 2:**
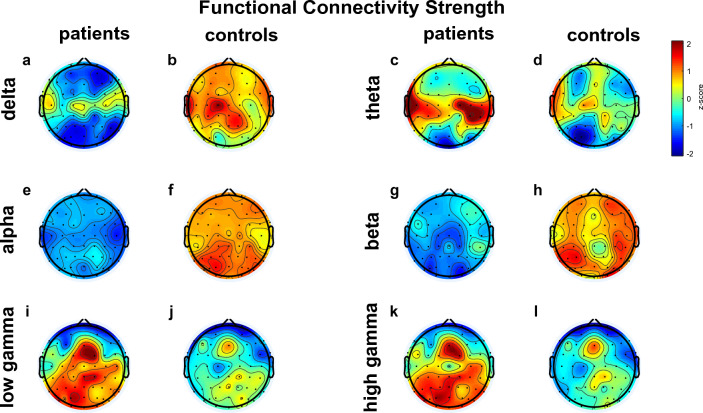
Resting-state functional connectivity. Topological representation of functional connectivity data (wPLI in delta and theta, and AEC-c in alpha, beta, low- and high gamma). HC = healthy controls, SCH = patients with schizophrenia.

Significantly diminished average functional connectivity was observed in patients compared to controls in the *delta* (*p*_corrected_ = 0.0474) and *alpha* (*p*_corrected_ = 0.0153) frequency ranges (Fig. 2) with medium to large effect sizes (for detailed results, see Table 2). On the other hand, there were no statistically significant differences in the strength of average functional connectivity in the *theta*, *beta*, *lower gamma* and *higher gamma* frequency bands.

**Table 2:** Differences between the group of patients and controls in functional connectivity strength, maximum betweenness centrality, degree centrality, diameter, leaf fraction, and assortativity.

Measure	Frequency	Statistic (t)	df	SCH versus HC	*p*-value	*p*_corrected_	Cohen's d	Conf. int (95%)	
Connectivity strength*	delta	**2.56**	**57.82**	**SCH < HC**	**0.0132**	**0.0474**	**0.653473**	**0.1219**	**1.1851**
	theta	− 0.79	54.79		0.4320	0.5760	− 0.19979	− 0.7191	0.3195
	alpha	**3.32**	**48.52**	**SCH < HC**	**0.0017**	**0.0153**	**0.878226**	**0.3359**	**1.4205**
	beta	1.33	47.12		0.1892	0.2961	0.353137	− 0.1689	0.8751
	gamma_1	− 1.02	49.62		0.3135	0.4341	− 0.25422	− 0.7743	0.2658
	gamma_2	− 1.62	48.34		0.1116	0.2009	− 0.40371	− 0.9269	0.1195
MST measures									
Maximum betweenness centrality	delta	− 1.92	57.97		0.0599	0.1198	− 0.49282	− 1.0186	0.0329
	theta	− 1.49	57.30		0.1418	0.2320	− 0.37922	− 0.9018	0.1434
	alpha	0.48	53.93		0.6315	0.7498	0.12603	− 0.3925	0.6445
	beta	0.46	56.34		0.6456	0.7498	0.119924	− 0.3985	0.6384
	gamma_1	**− 2.96**	**57.23**	**SCH > HC**	**0.0045**	**0.0270**	**− 0.76427**	**− 1.3008**	**− 0.2278**
	gamma_2	− 1.30	57.77		0.1974	0.2962	− 0.33294	− 0.8545	0.1886
Maximum degree centrality	delta	**− 2.60**	**53.32**	**SCH > HC**	**0.0120**	**0.0474**	**− 0.65448**	**− 1.1861**	**− 0.1229**
	theta	− 2.31	53.87	SCH > HC	0.0248	0.0634	− 0.58131	− 1.1101	− 0.0525
	alpha	− 0.26	57.66		0.7977	0.8297	− 0.06569	− 0.5838	0.4524
	beta	− 0.39	55.38		0.7001	0.7876	− 0.10076	− 0.6191	0.4176
	gamma_1	− 2.37	57.37	SCH > HC	0.0209	0.0628	− 0.6047	− 1.1343	− 0.0751
	gamma_2	− 1.10	57.02		0.2739	0.3945	− 0.28577	− 0.8064	0.2348
Diameter	delta	**3.33**	**55.41**	**SCH < HC**	**0.0016**	**0.0153**	**0.865735**	**0.3241**	**1.4073**
	theta	**2.69**	**57.32**	**SCH < HC**	**0.0093**	**0.0474**	**0.695235**	**0.1619**	**1.2286**
	alpha	− 0.21	57.81		0.8352	0.8352	− 0.05381	− 0.5719	0.4643
	beta	− 0.69	55.38		0.4954	0.6342	− 0.17857	− 0.6976	0.3405
	gamma_1	2.49	52.95	SCH < HC	0.0161	0.0526	0.651196	0.1197	1.1827
	gamma_2	0.66	52.93		0.5109	0.6342	0.173347	− 0.3456	0.6923
Leaf fraction	delta	**− 3.61**	**57.95**	**SCH > HC**	**0.0006**	**0.0115**	**− 0.92744**	**− 1.4725**	**− 0.3824**
	theta	**− 2.96**	**57.96**	**SCH > HC**	**0.0045**	**0.0270**	**− 0.7576**	**− 1.2938**	**− 0.2214**
	alpha	− 0.29	57.33		0.7760	0.8297	− 0.07383	− 0.5920	0.4443
	beta	− 0.25	57.66		0.8067	0.8297	− 0.06337	− 0.5815	0.4548
	gamma_1	− 2.10	51.63	SCH > HC	0.0403	0.0908	− 0.55238	− 1.0801	− 0.0246
	gamma_2	− 1.80	45.22		0.0780	0.1478	− 0.47975	− 1.0051	0.0456
Assortativity	delta	**3.79**	**54.54**	**SCH < HC**	**0.0004**	**0.0115**	**0.957137**	**0.4104**	**1.5039**
	theta	2.28	56.68	SCH < HC	0.0264	0.0634	0.578711	0.0500	1.1074
	alpha	2.33	52.24	SCH < HC	0.0236	0.0634	0.611517	0.0816	1.1414
	beta	**2.57**	**56.02**	**SCH < HC**	**0.0130**	**0.0474**	**0.650158**	**0.1187**	**1.1816**
	gamma_1	1.58	56.66		0.1190	0.2040	0.410128	− 0.1133	0.9335
	gamma_2	1.99	57.64		0.0514	0.1089	0.512795	− 0.0136	1.0392

*wPLI in case of delta and theta frequency ranges, AEC-c in case of alpha, beta, low and high gamma.

Significant values are in bold.

### MST parameters

#### Diameter

**Figure 3:**
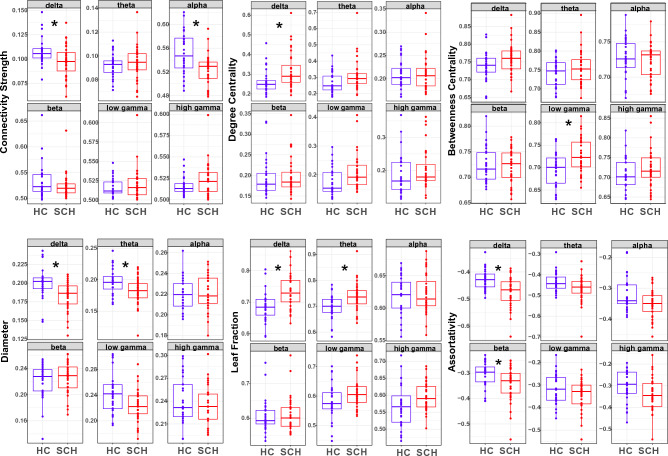
Between-group differences in average resting-state functional connectivity strength and measures of functional network structure. HC = healthy controls, SCH = patients with schizophrenia. * *p*_corrected_ < 0.05.

MST diameter was lower in the group of patients in *delta* (*p*_corrected_ = 0.0153), and *theta* (*p*_corrected_ = 0.0474) frequency bands, it was also lower in case of *lower gamma* but the difference was only significant on a trend level after FDR correction (*p*_corrected_ = 0.0526). No statistically significant differences were found in the *alpha*, *beta*, and *high gamma* frequency bands (Table 2**, **Fig. 3). For a topological representation of the average MST-s please see Figures S1, S2, and S3 in the Supplementary Information.

#### Leaf fraction

MST leaf fraction was higher in the group of patients in the *delta* (*p*_corrected_ = 0.0115) and *theta* (*p*_corrected_ = 0.027) frequency bands. In *low gamma* band a similar difference was observed, but it did not remain statistically significant after FDR correction (*p*_corrected_ = 0.0908). Differences in leaf fraction between the two group were not significant in the *alpha*, *beta*, and *high gamma* frequency bands (Table 2**, **Fig. 3).

#### Maximum degree centrality

MST maximum degree centrality was significantly higher in patients in *delta* (*p*_corrected_ = 0.0474) band. After FDR correction, the difference in the *theta* (*p*_corrected_ = 0.0634) and *lower gamma* (*p*_corrected_ = 0.0628) remained significant on a trend level. Maximum degree centrality did not differ significantly between the study groups in *alpha*, *beta*, and *higher gamma* frequency bands (Table 2**, **Fig. 3).

#### Maximum betweenness centrality

MST maximum betweenness centrality was higher in the group of patients compared to controls in the *lower gamma* frequency band (*p*_corrected_ = 0.027). The differences, however were not statistically significant in *delta*, *theta*, *alpha*, *beta*, and *high gamma* frequency bands (Table 2**, **Fig. 3).

#### Assortativity

Assortativity was lower (i.e. dissasortativity was higher, meaning that high-degree nodes were more likely to attach to low-degree nodes) in the group of patients in the *delta* (*p*_corrected_ = 0.0115) and *beta* (*p*_corrected_ = 0.0474) frequency bands. The differences in *theta*, *alpha*, and *lower* and *higher gamma* band were not statistical significance (Table 2**, **Fig. 3).

### Further analyses

#### Mean betweenness centrality

Patients showed higher maximum betweenness centrality in *lower gamma* while the mean betweenness centrality tended to be lower in the patient group (See Table 2 and Table 3).

**Table 3:** Differences between patients with schizophrenia and controls in mean betweenness centrality.

Frequency	Region	Statistic (t)	SCH vs. HC	df	*p*-value	Cohen's d
Mean Betweenness Centrality						
delta	**Global**	**3.06**	**SCH < HC**	**56.64**	**0.0034**	**0.7927**
	**Anterior**	**2.14**	**SCH < HC**	**54.60**	**0.0372**	**0.5571**
	**Posterior**	**2.44**	**SCH < HC**	**57.84**	**0.0176**	**0.6288**
theta	**Global**	**2.42**	**SCH < HC**	**57.98**	**0.0187**	**0.6213**
	**Anterior**	**2.20**	**SCH < HC**	**57.43**	**0.0316**	**0.5686**
	Posterior	1.88		58.00	0.0652	0.4820
alpha	Global	− 1.31		57.54	0.1962	− 0.3373
	Anterior	− 1.85		55.20	0.0701	− 0.4809
	Posterior	0.93		56.67	0.3579	0.2353
beta	Global	− 0.67		52.52	0.5070	− 0.1751
	Anterior	− 1.76		57.29	0.0843	− 0.4538
	Posterior	1.53		57.54	0.1320	0.3895
gamma_1	**Global**	**2.61**	**SCH < HC**	**51.93**	**0.0119**	**0.6842**
	Anterior	1.79		50.39	0.0790	0.4722
	Posterior	1.77		53.61	0.0817	0.4638
gamma_2	Global	0.71		54.31	0.4780	0.1865
	Anterior	2.00		48.50	0.0514	0.5281
	Posterior	− 0.69		57.42	0.4924	− 0.1784

Significant values are in bold.

In *delta* (*p* = 0.0034), *theta* (*p* = 0.0187) and as well as in *lower gamma* (*p* = 0.0119) frequency bands, *global* average betweenness centrality was lower in patients with schizophrenia compared to controls. In case of *alpha*, *beta* and *higher gamma*, between-group differences were not statistically significant (Table 3).

As far as the anterior region is concerned, differences between the two groups within the *delta* (*p* = 0.0372) and *theta* (*p* = 0.0316) frequency bands were statistically significant, but in case of *alpha*, *beta*, *lower* and *higher gamma*, the differences failed to reach statistical significance.

Concerning the posterior region, between-group difference in mean betweenness centrality was statistically significant in the *delta* frequency band (*p* = 0.0176) but differences in *theta*, *alpha*, *beta*, *lower gamma* and *higher gamma* frequency ranges were not statistically significant.

## Discussion

In this study we aimed to examine the strength of global average functional connectivity and functional network topology in patients with schizophrenia and healthy controls in resting state. Based on previous results from Alzheimer’s research^41^, functional connectivity strength between each channel was computed with wPLI in lower frequencies (delta and theta), and AEC-c in higher frequencies (alpha, beta, low and high gamma). For the analysis of network topology, MST algorithms were applied.

We found weaker global average functional connectivity in delta and alpha frequency bands in patients, compared to controls. Whereas no significant between-group differences were found in case of the theta, beta, lower and higher gamma frequency bands.

Although some previous findings indicate decreased functional connectivity strength in lower frequency bands in general, and increased connectivity in higher frequencies^19^, contradicting results also exist, e.g. Krukow and colleagues^11^ reported increased average connectivity in theta and decreased connectivity in lower alpha, while no differences in other frequency bands were found in the study. It can be seen however that results concerning lower frequencies (delta and theta) are less conclusive, and could be a affected by methodological factors^2,8,9,11,17^, on the other hand, decreased functional connectivity within the alpha range has been found to be weaker in patients in the majority of studies irrespective of methodological differences and demographic or clinical variation^8,11,16,17,19^.

Our findings are in accordance with most results previously reported in the literature. As we hypothesized, a particularly robust between-group difference was found in the alpha band (patients < controls). Functional connectivity within the alpha band can be particularly important in large-scale communication between distant cortical regions^46,47^. Alpha activity is related to the coordination of information flow both within and between different functional brain networks^46,48^. Previous results on resting-state brain activity indicate that during rest, active information processing takes place, the default mode network is highly activated, associated with inward attention, self-reflection, autobiographical memory^49^. Furthermore, research indicates that alpha activity is related to the somatosensory network, and the decoupling of the default mode network and networks related to external attention and external information processing^46^.

Weaker functional connectivity in the alpha band was also observed in other conditions, such as autism spectrum disorder^46^; and, interestingly, sleep deprivation—that is often used as a model of schizophrenia and psychosis^50–52^—was found to be related to decreased alpha band connectivity in a widespread network^47^.

Recent data suggest that delta activity can be related to resting state functional MRI connectivity^53^, which is also found to be decreased in schizophrenia^20,21^. In our study, functional connectivity in the delta band also differed between the two groups (patients < controls). Past research indicate that connectivity in the delta band is closely related to long-range cortico-cortical connectivity^53^. In particular, functional connectivity in the 2–5 Hz range was found to be a marker of conscious states^54^.

Overall, our finding of decreased connectivity in alpha and delta band could be related to disrupted intra- and inter-network communication during rest, reflecting mild alteration of consciousness, self-perception, altered sensory processing, inward and outward attention, and attention instability (that is closely related to cognitive deficits in schizophrenia)^46,47,53,54^.

We did not find between-group differences in connectivity strength within higher frequency bands (beta, and low and high gamma), but it can be seen that past findings regarding differences in functional connectivity strength between patients of schizophrenia and healthy controls in higher frequency bands are less conclusive and more ambiguous in general^2,8,9,13,14,17^.

Conventional graph theoretical measures are highly influenced by the number of connections and the strength of connectivity^23^. Consequently, lower connectivity strength in the patient group could have led to biased results^20^. In order to overcome the potential bias, we decided to use the MST method that has been shown to be more robust and much less likely to be influenced by different factors, such as the strength of average functional connectivity^25^.

In a resting state fMRI study van Dellen and colleagues^20^ have demonstrated lower global average functional connectivity strength in patients with schizophrenia spectrum disorder but no differences in MST network properties were found in schizophrenia as opposed to subjects with bipolar disorder, where the network was less integrated. The authors concluded that the neural correlates of psychosis might be different in the two psychiatric conditions; and past findings of network topology alterations in schizophrenia were most likely attributable to methodological issues arising from the sensitivity of conventional graph theoretical measures to the average connectivity strength. Contrary to their findings however, results of more recent studies using the MST method report a tendency toward *over-centralization* and increased randomness in terms of increased network *disassortativity* in patients with schizophrenia^10,11,34^.

In accordance with more recent data on the subject, we have also found significant between-group differences in the most frequently analyzed MST measures. Our results indicate *increased centralization* in the group of patients, as we found lower diameter (in delta and theta), higher leaf fraction (in delta and theta), higher maximum betweenness centrality (in low gamma), and higher maximum degree centrality (in delta and theta).

The closest study to this paper has been done by Krukow and colleagues^11^, who investigated network structure with MST besides resting-state EEG functional connectivity strength in multiple frequency bands in first-episode schizophrenia patients. As a connectivity measure, they used phase-lag index. Concerning global MST metrics, the study found similar results to us. Their results include lower diameter in delta, beta, and gamma; higher leaf fraction in delta and gamma, higher maximum betweenness centrality in beta. Although, differences were not always statistically significant in the same frequency bands as in our study (perhaps partly due to some methodological differences), the overall findings of the two studies still point in the same direction: increased global integration in patients compared to controls.

Along with Krukow and colleagues^11^ (who reported higher disassortativity in patients in the delta band), we found that the network of patients was more dissassortative, more random in a sense, meaning that high-degree nodes were far more likely to attach to low-degree nodes, so the likelihood of prominent hubs connecting to each other was significantly lower in the patient group. As a consequence, rich clubs can hardly be formed^11^. Assortativity is also used as a measure of resilience, as disassortative networks are more vulnerable to hub failures^43^.

Our results on global MST metrics are also in accordance with fMRI research. For example, Liu and colleagues^34^ found a more star-line global network structure, increased integration (lower path length, higher leaf fraction, higher maximum degree centrality [significant only on a trend level], but no difference in assortativity) in subjects with schizophrenia. Alexander-Bloch and colleagues^22^—examining network topology of patients with childhood onset schizophrenia—found increased global efficiency, lower clustering, and decreased modularity in patients compared to controls.

When the optimal balance between local segregation and global integration processes (referred to as small-worldness) gets disturbed, and the network becomes biased towards integration, it leads to less efficient network organization. Information transfer becomes heavily reliant on a few number of highly connected nodes and hubs with high betweenness centrality. As prominent nodes are directly connected to many leafs, they can become over-connected and over-activated, and eventually fail^27^. Global over-integration hinders selective information processing, resulting in the breakdown of the hierarchical network structure and the boundaries between functionally specialized systems^22^.

Although, the exact neurological mechanisms leading to altered functional connectivity and disturbed network topology in schizophrenia are not fully understood, some authors link these alterations to abnormal brain developmental processes related to the disease: abnormal axonal growth, synaptic pruning, and white matter development^22^.

At the same time, literature shows that network structure in (healthy) humans is not stable throughout the lifetime. The process of aging is characterized by somewhat similar changes in brain network topology as those found in schizophrenia: network structure of the aging brain becomes more star-like, more globally integrated with less functional specification^34^. Some data suggest that symptom severity in schizophrenia can be related to age-related changes in network structure^34^. Jonak and colleagues^10^ compared MST metrics between first episode and multiepisode patients with schizophrenia, and found that the increase in integration was associated with the longer illness duration. The mechanism behind functional network imbalances in schizophrenia is often explained in the literature by the cascading network failure hypothesis^55^, borrowed from dementia research, according to which the redistribution of the workload of dysfunctional nodes may lead to over-centralization^23,26^.

The literature is not consistent in the question whether hub nodes—nodes important in global communication—are shifted to more anterior or posterior regions in schizophrenia. Liu and colleagues^34^ found more hubs in frontal regions but Jonak et al.^10^ and Krukow et al.^11^ presented evidence of defrontalization in terms of relatively weakened importance of frontal regions in global communication in patients with schizophrenia. Krukow and colleagues^11^ reported higher average betweenness connectivity in the posterior area in delta and gamma frequency bands in patients. We could not replicate this result. Concerning regional differences, we found no evidence of either defrontalization or increased frontalization in patients, as mean betweenness centrality (indicating average global hub importance) appeared to be lower in general in patients irrespective of the examined area (anterior or posterior). Interestingly, global average betweenness centrality was lower in patients (in delta, theta, and low gamma), while at the same time, maximum betweenness centrality was higher (in low gamma). In this respect, however, our results closely resemble those obtained by Krukow and colleagues^11^, who found higher maximum betweenness centrality along with lower mean betweenness centrality in the beta band in patients. The authors interpreted the result as an indication of imbalance in hub strength.

Besides its strengths, our study has a number of potential limitations as well. We made our conclusions on the basis of a very limited amount of data, i.e. seven 8 s long segments of resting state EEG per subject were analyzed. However, we more or less were still able to replicate the results of some previous studies using a higher number of epochs (e.g. Krukow et. al^11^ analyzed thirty 8 s long segments per subject).

Although, we have taken the necessary steps to eliminate artifacts from the recordings, there was no Faraday cage in the EEG recording setup. This fact may weaken the validity of our results of the gamma frequency band. Future research is needed to replicate our results with more advanced equipments.

It also has to be admitted that, although the MST approach has a number of advantages over conventional graph analytical measures, it is not devoid of limitations^24^. Since between-group differences in average functional connectivity strength were found, conventional graph analysis methods would more likely yield misleading results. MST, on the other hand, is an unbiased network representation^24^, however, as it is a simplified subnetwork of the original network. Nevertheless, bias towards over-centralization in the group of patients indirectly suggests a violation from small-wordness, and deficient intra-network communication^11^.

Another limitation of our research could be the low sample size that did not allow us to perform subgroup analyses, although e.g. evidence exist of disease duration being a possible influential factor in functional network topology deviations in schizophrenia^34^. Furthermore, as far as schizophrenia is a complex and diverse mental disease, comparing network topology of patients with different types of the disease could also be beneficial.

In summary, weaker average functional connectivity was found in two frequency bands (delta and alpha) in patients, compared to controls. Our results on functional network topology indicate increased centralization, increased global integration in the group of patients. The network of patients was more disassortative, more vulnerable: high-degree nodes were more likely to connect to low-degree nodes preventing the formation of rich clubs. Excessive integration processes can lead to overload and failure of central hubs. These results together can indicate a breakdown of the modular network structure in patients with schizophrenia, somewhat comparable to the data found in aging and dementia research.

### Supplementary Information

Supplementary Information.

## Data Availability

The data that support the findings of this study are available from the corresponding author upon reasonable request.
