# Supplementary material for: Minimum spanning tree analysis of EEG resting-state functional networks in schizophrenia
Source: Sci Rep. 2024 May 7;14:10495. doi: 10.1038/s41598-024-61316-8 (PMC11076461; doi:10.1038/s41598-024-61316-8)
Supplement: Supplementary file 1 — Supplementary Information. [file 41598_2024_61316_MOESM1_ESM.pdf]

Supplementary Information

Minimum spanning tree analysis of EEG resting-state functional networks in schizophrenia

Melinda Becske, Csilla Marosi, Hajnalka Molnár, Zsuzsanna Fodor, Kinga Farkas, Frigyes Sámuel Rácz, Máté Baradits, Gábor Csukly

The figures below represent the most frequently occurring MST connections in the two groups. The figures were constructed based on the group averaged MST adjacency matrices with the BrainNet toolbox (https://www.nitrc.org/projects/bnv/). Only the strongest connections are shown for clarity (threshold for delta and theta is 0.057, for the rest of the frequencies is 0.07). The size of each node is proportional to the average degree of the given node, and the thickness of the edges are proportional to the average strength of the connection (thick lines indicate that the given connection was part of the MST in more cases) [1].

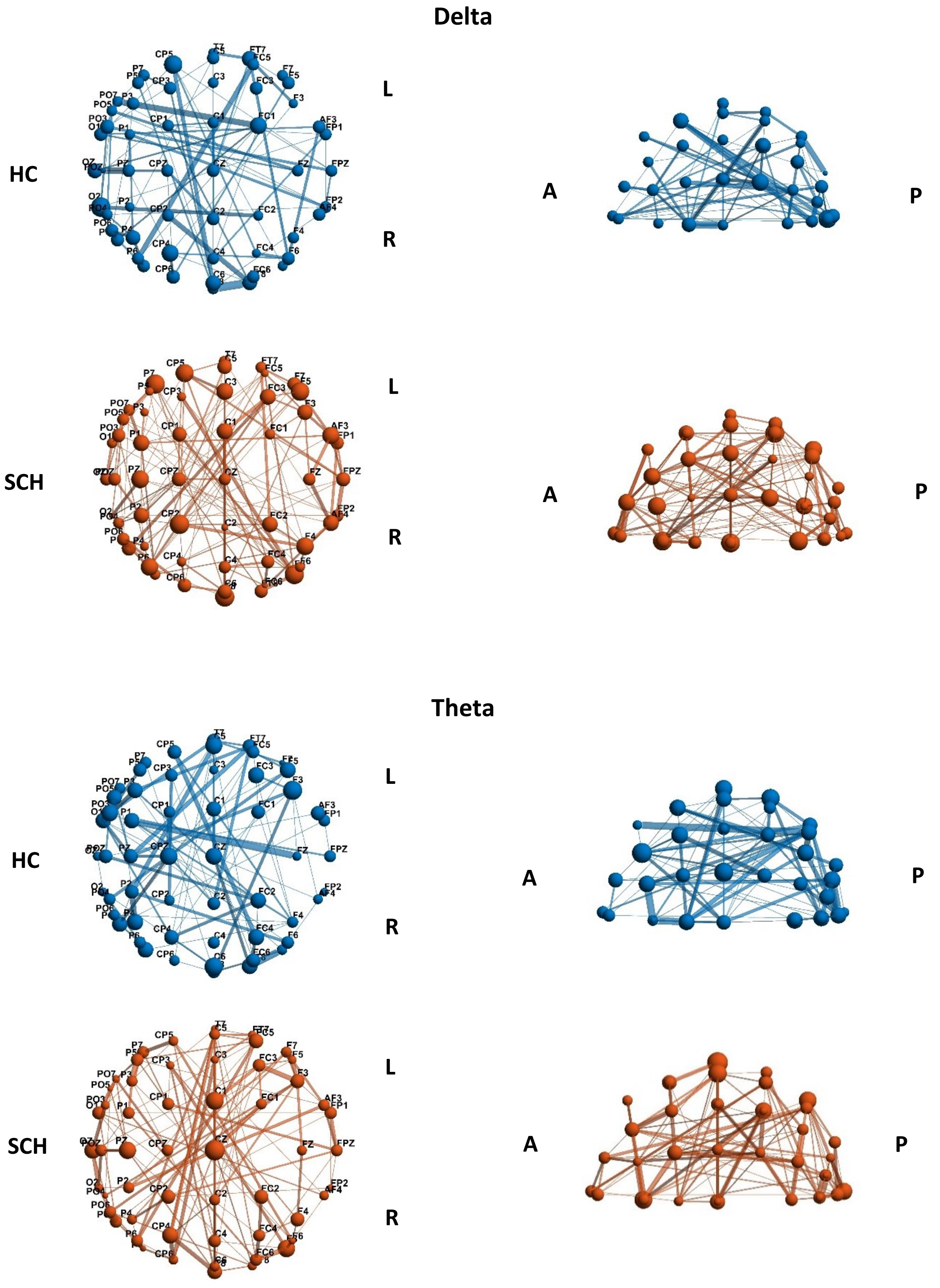

Figure S1. The most important connections based on the averaged MST adjacency matrices in the delta and theta frequency bands (HC = healthy controls, SCH = patients with schizophrenia, L = left, R = right, A = anterior, P = posterior)

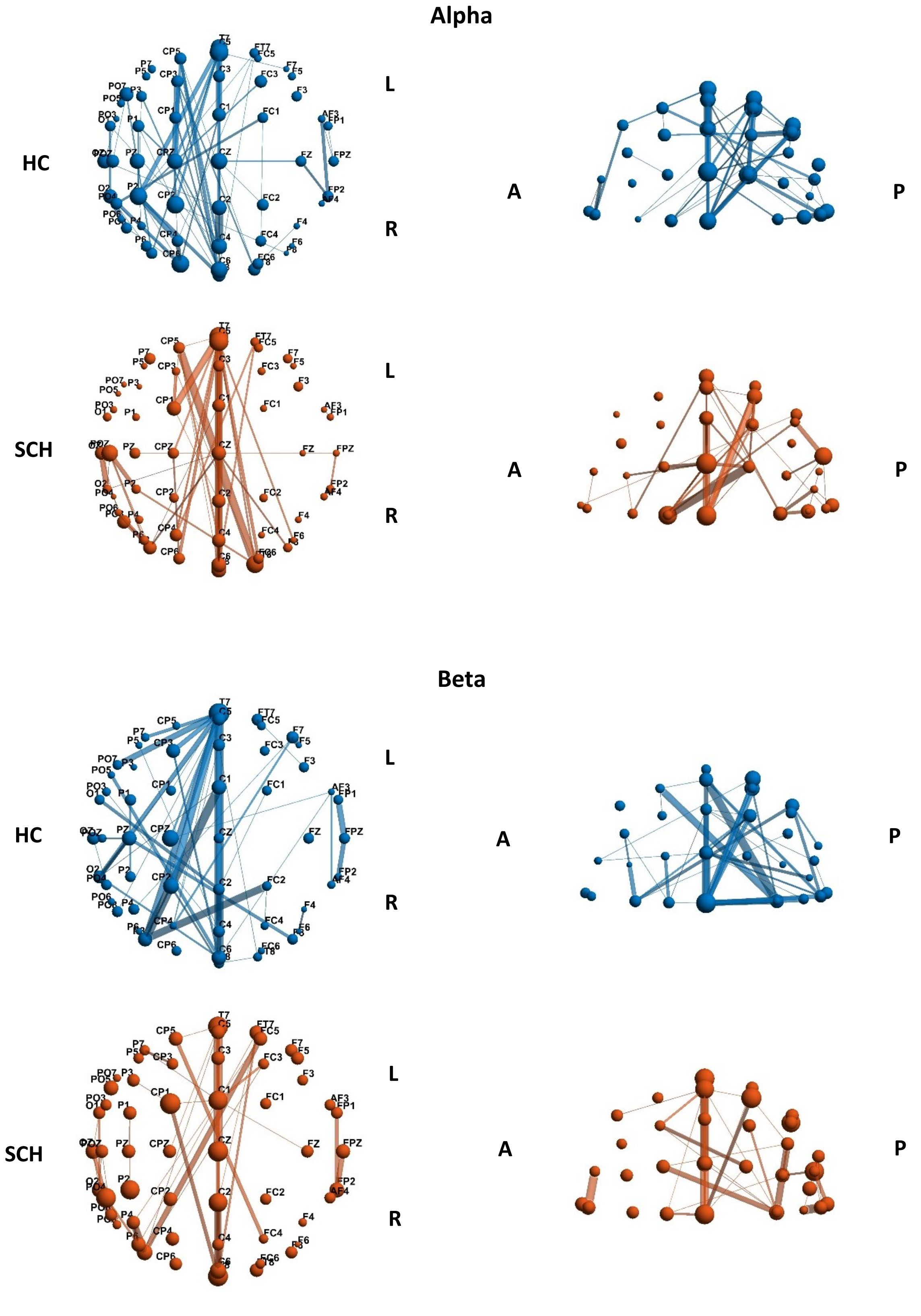

Figure S2. The most important connections based on the averaged MST adjacency matrices in the alpha and beta frequency bands (HC = healthy controls, SCH = patients with schizophrenia, L=left, R=right, A=anterior, P=posterior)

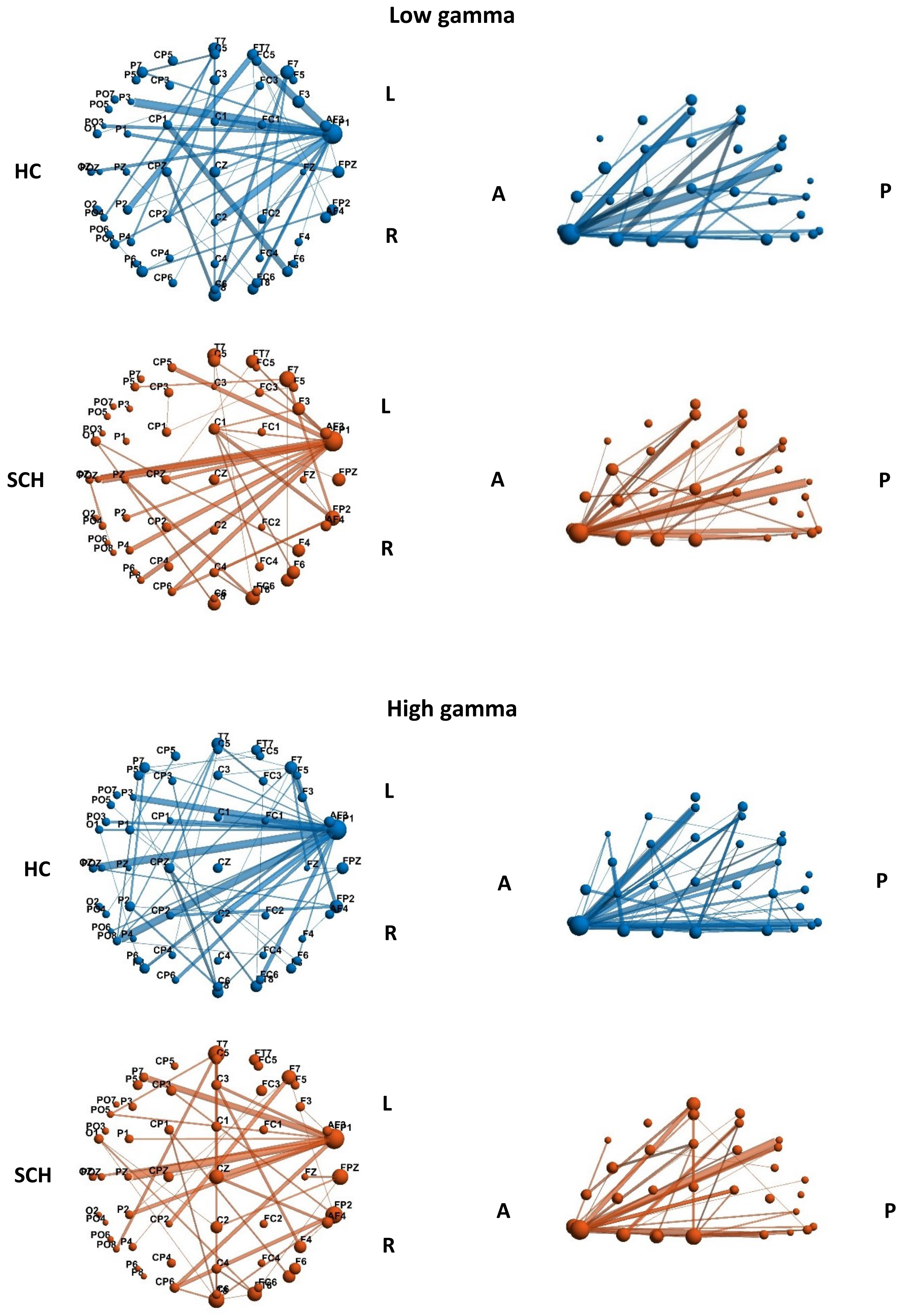

Figure S3. The most important connections based on the averaged MST adjacency matrices in the low and high gamma frequency bands (HC = healthy controls, SCH = patients with schizophrenia, L=left, R=right, A=anterior, P=posterior)

References

1. van Dellen, E. *et al.* Minimum spanning tree analysis of the human connectome. *Hum. Brain Mapp.* **39**, 2455–2471 (2018).
